# The 2014 Ebola virus disease outbreak in Pujehun, Sierra Leone: epidemiology and impact of interventions

**DOI:** 10.1186/s12916-015-0524-z

**Published:** 2015-11-26

**Authors:** Marco Ajelli, Stefano Parlamento, David Bome, Atiba Kebbi, Andrea Atzori, Clara Frasson, Giovanni Putoto, Dante Carraro, Stefano Merler

**Affiliations:** Bruno Kessler Foundation, Trento, Italy; District Medical Officer, Pujehun District, Ministry of Health and Sanitation, Freetown, Sierra Leone; Medical Superintendent Pujehun Hospital, Ministry of Health and Sanitation, Freetown, Sierra Leone; Doctors with Africa – CUAMM, Padova, Italy

**Keywords:** Computational model, Ebola, Key time periods, Transmissibility, Transmission chain

## Abstract

**Background:**

In July 2014, an outbreak of Ebola virus disease (EVD) started in Pujehun district, Sierra Leone. On January 10th, 2015, the district was the first to be declared Ebola-free by local authorities after 49 cases and a case fatality rate of 85.7 %. The Pujehun outbreak represents a precious opportunity for improving the body of work on the transmission characteristics and effects of control interventions during the 2014–2015 EVD epidemic in West Africa.

**Methods:**

By integrating hospital registers and contact tracing form data with healthcare worker and local population interviews, we reconstructed the transmission chain and investigated the key time periods of EVD transmission. The impact of intervention measures has been assessed using a microsimulation transmission model calibrated with the collected data.

**Results:**

The mean incubation period was 9.7 days (range, 6–15). Hospitalization rate was 89 %. The mean time from the onset of symptoms to hospitalization was 4.5 days (range, 1–9). The mean serial interval was 13.7 days (range, 2–18). The distribution of the number of secondary cases (*R*_*0*_ = 1.63) was well fitted by a negative binomial distribution with dispersion parameter *k* = 0.45 (95 % CI, 0.19–1.32). Overall, 74.3 % of transmission events occurred between members of the same family or extended family, 17.9 % in the community, mainly between friends, and 7.7 % in hospital. The mean number of contacts investigated per EVD case raised from 11.5 in July to 25 in September 2014. In total, 43.0 % of cases were detected through contact investigation. Model simulations suggest that the most important factors determining the probability of disease elimination are the number of EVD beds, the mean time from symptom onset to isolation, and the mean number of contacts traced per case. By assuming levels and timing of interventions performed in Pujehun, the estimated probability of eliminating an otherwise large EVD outbreak is close to 100 %.

**Conclusions:**

Containment of EVD in Pujehun district is ascribable to both the natural history of the disease (mainly transmitted through physical contacts, long generation time, overdispersed distribution of secondary cases per single primary case) and intervention measures (isolation of cases and contact tracing), which in turn strongly depend on preparedness, population awareness, and compliance. Our findings are also essential to determine a successful ring vaccination strategy.

**Electronic supplementary material:**

The online version of this article (doi:10.1186/s12916-015-0524-z) contains supplementary material, which is available to authorized users.

## Background

The 2014–2015 Ebola virus disease (EVD) epidemic in West Africa was first detected in March 2014 in Guinea [1]. With a total of 28,220 reported cases and 11,291 reported deaths (as reported by the WHO on September 16, 2015), the largest EVD outbreak ever documented [2, 3], the disease showed its devastating potential. In July 2014, a local outbreak started in Pujehun district, Sierra Leone, and on January 10^th^, 2015, the district was the first to be declared Ebola-free by local authorities. The outbreak affected a very isolated region and, in particular, two small, strictly connected areas, namely Zimmi (a rural town) and Dumagbe (a village).

Early detection of cases, isolation, contact tracing, safe burials, population awareness, and compliance, all critical factors for mitigating or containing EVD, were implemented during the course of the epidemic in West Africa, albeit with different degrees of success. Nevertheless, the quantitative evaluation of their impact is still under debate. Most of the analyses conducted so far are based on time series of cases and on the scarcely available information about both local characteristics of EVD transmission and implemented intervention measures [4–7].

The EVD outbreak in Pujehun is considered a nearly unique example of a successfully contained outbreak in a rural and geographically isolated district. By combining epidemiological investigation and modeling techniques we aim to reconstruct the main characteristics of the outbreak and to evaluate the impact of the implemented intervention measures. This allows us to clarify the reasons behind the successful local containment of the epidemic.

## Methods

This study was conducted in Pujehun District, in the Southern Province of Sierra Leone and with a population of approximately 375,000 inhabitants. The district has one of the lowest population densities of Sierra Leone, with most people living in villages of less than 2,000 residents. The local healthcare system consists of one Government District Hospital with an 87-bed capacity and 74 Peripheral Health Units. Two Ebola Holding Centers (EHCs) were operative by the end of June 2014, one inside the Pujehun hospital, later moved outside the town, and the second one in Zimmi. The Sierra Leone Ethics and Scientific Review Committee approved the study protocol in July 2015. Collected data consisted of routine health data and medical records, were encrypted and anonymous, and did not contain any information that might be used to identify individual patients; therefore, the study did not require informed consent.

### Transmission chain

We reconstructed the transmission chain in Pujehun district by analyzing the registers of the two EHCs, contact tracing forms, and by interviewing healthcare workers (HCWs) involved in the management of the outbreak (three of them being co-authors of this study), survived case patients, and relatives of deceased case patients. We considered both confirmed and probable cases. Data on age, sex, date of symptom onset, hospital admission, and death/discharge were obtained from district health management team and registers of the two EHCs. An initial transmission chain was obtained by analyzing contact tracing forms (reporting the name of the contacts of primary cases, the relation between primary case and contacts, the date of the last contact, and the type of contact), available for 21 case patients. We filled the gaps in the initial transmission chain by discussing with authors CF, DB, and AK, who managed the two EHCs in Pujehun district during the outbreak and by discussing with survived case patients and relatives of deceased case patients – to this purpose, author GP visited the two villages at the end of the outbreak. No written informed consent was collected. Case patients and their relatives orally agreed to the interview. Information on individual patients has been anonymized for analysis, presentation, and dissemination of theresults. A contact tracing form example is available in Additional file 1.

### Key time periods

We investigated the key times regulating infection transmission by analyzing the registers of the two EHCs (Additional file 2) and contact tracing forms. We estimated (1) the incubation period, which is the time from exposure to symptom onset (it is computed as the time from the last contact with the infector to symptom onset); (2) the time from symptom onset to hospitalization, a measure of the transmission period in the community for isolated case patients; (3) the time from symptom onset to death for unhospitalized cases (the term unhospitalized refers to EVD cases that died before or soon after arriving in the EHC of Pujehun district), a measure of the transmission period in the community for non-isolated cases; (4) the time from hospitalization to discharge or death, which are indicative of the number of EVD beds required for timely isolation of cases; (5) the serial interval, which is the time between symptom onset in a primary infector and in secondary cases. In this analysis we discarded all missing data by assuming that they were missing at random.

### Reproduction numbers

We analyzed the distribution of the number of secondary cases generated by all cases and we inferred estimates of the basic reproduction number *R*_*0*_, which is the mean number of secondary cases generated by an index case introduced in a fully susceptible population. *R*_*0*_ gives insight into the effort required to control the disease and when the reproduction number is below 1, the infection cannot be sustained. We also estimated the net reproduction number, *R*_*t*_, which describes changes of *R*_*0*_ over time due to variations of the transmissibility (e.g. behavioral changes of the population, effect of interventions). We estimated *R*_*t*_ over time from the time series of the exposure times of case patients and from our estimate of the serial interval.

### Interventions

We gathered information on interventions by interviewing HCWs involved in the management of the outbreak and by analyzing contact tracing forms. We estimated the probability of hospitalization of EVD cases, the percentage of cases detected and isolated through contact investigation over time, and the probability of community burials over time. Traditional burials in Sierra Leone consist of a set of practices, including washing, touching or kissing the body of the deceased, which may promote infection transmission. Herein, we use the term community burials as opposed to safe burials, these being conducted by EVD burial teams following specific procedures (starting from the moment the teams arrive in the village up to their return to the hospital or team headquarters after burial and disinfection procedures) aimed at minimizing the risk of infection transmission during burial ceremonies. Of note, community burials may be either safe or unsafe depending on several factors, e.g. population awareness.

### Impact of interventions in Pujehun

We developed a microsimulation model of EVD transmission for Pujehun district. The model is an individual-based model similar to that used to describe the transmission of EVD in Liberia [7]. The progression of the disease is based on our estimates of the key time periods. The model accounts for all implemented intervention measures, namely hospital isolation of cases, safe burials, and contact tracing. Hospitalization triggers contact investigation and deceased hospitalized case patients are safely buried. Transmission parameters were estimated by assuming our estimates about probability of hospitalization, community burial, and contact tracing levels. The impact of all implemented interventions was estimated through a detailed sensitivity analysis.

Further methodological details are reported in Additional file 3.

## Results

### The outbreak

**Fig. 1:**
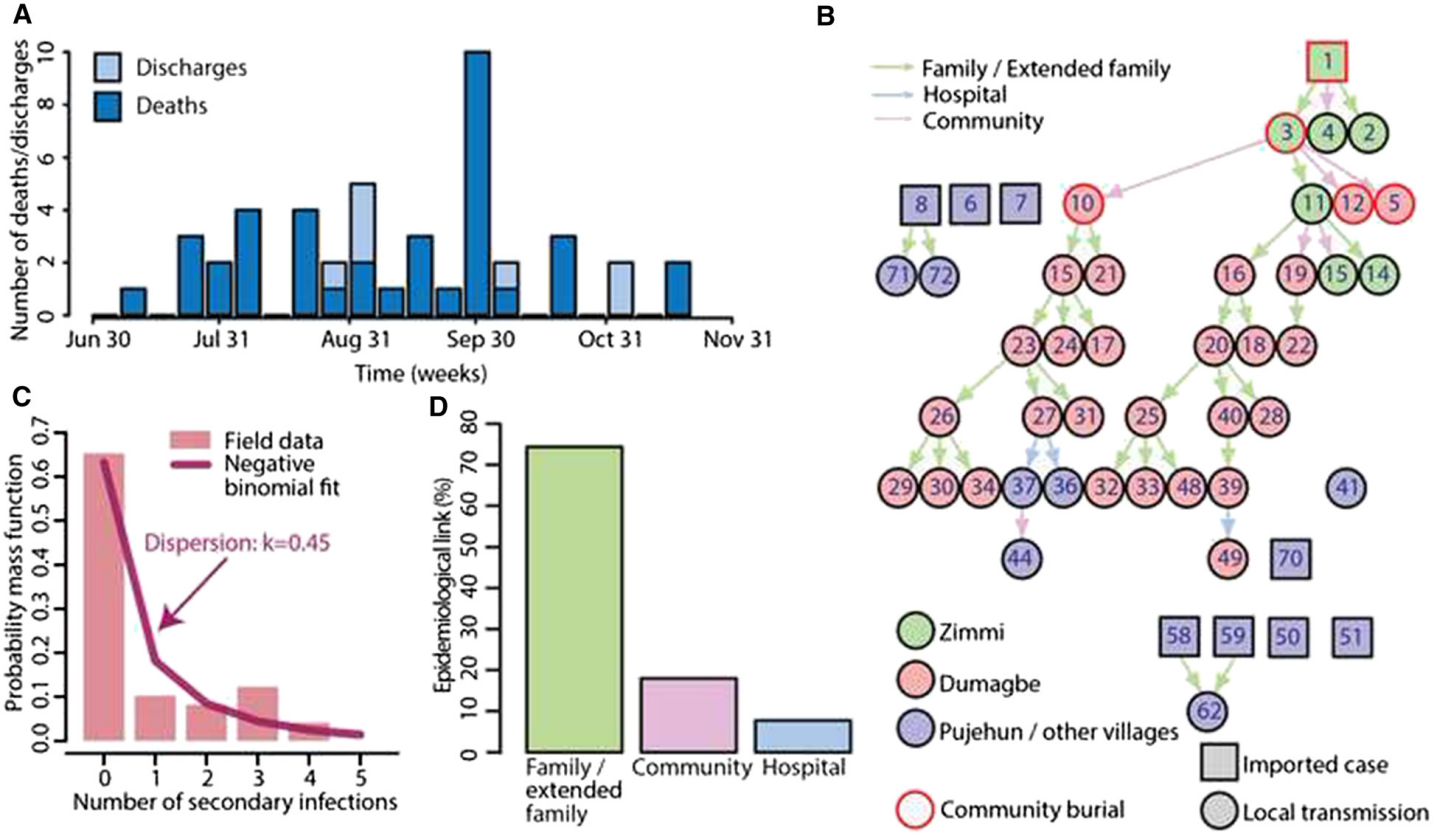
Ebola virus disease (EVD) transmission chain in Pujehun district. (**a**) Number of deaths/discharges over time. (**b**) Transmission chain of the 2014 EVD outbreak in Pujehun district, Sierra Leone. Symbols are defined in the figure. Numbers are patient ID (see Additional file 2 for detailed information on case patients). (**c**) Distribution of the number of secondary cases generated by EVD cases. The line represents the negative binomial fit. (**d**) Percentage of transmission events in different settings

A total of 49 case patients, consisting of 31 confirmed and 18 probable cases, were registered between July 2014 and November 2014 in Pujehun District (Table 1). Of these, 19 (38.8 %) were male and 30 (61.2 %) were female. Mean age was 31.7 years (range, 3–85), and 14 case (28.6 %) patients were aged ≤15. The case fatality rate was 85.7 % (42/49, 95 % CI, 72.7–94.1). The time series of cases is shown in Fig. 1a.

**Table 1:** Characteristics of probable and confirmed cases of Ebola virus disease in Pujehun district, Sierra Leone

	Number (%)
Children (0–15 years old)	8/49 (16.3 %)
Adults (16–64 years old)	35/49 (71.4 %)
Females	30/49 (61.2 %)
Healthcare workers (HCW)	3/49 (6.1 %)
Admitted to hospital	44/49 (88.8 %)
Deaths	42/49 (85.7 %)
Community burials	5/42 (11.9 %)
Cases imported from abroad	9/49 (18.4 %)
In the local transmission chains	39/40 (97.5 %)
Exposed in family/extended family	29/39 (74.3 %)
Exposed in the community	7/39 (17.9 %)
Exposed in hospital (patients)	1/39 (2.6 %)
Exposed in hospital (HCWs)	2/39 (5.1 %)
Touched the body fluids of the case (blood, vomit, saliva, urine, feces)	6/31 (19.4 %)
Had direct physical contact with the body of the case (alive or dead)	16/31 (51.6 %)
Touched or shared the linen, clothes, or dishes/eating utensils of the case	23/31 (74.2 %)
Slept, ate, or spent time in the same household or room as the case	20/31 (64.5 %)

### Key time periods

The mean incubation period was 9.7 days (range, 6–15 days). The mean time from the onset of symptoms to hospitalization was 4.5 days (range, 1–9 days), with no statistically significant changes over time. The mean time to death after admission to the hospital was 3.1 days (range, 0–8 days), and the mean time to discharge was 7.0 days (range, 3–12 days). The mean time to death after symptom onset was 6.6 days (range, 0–22 days) for hospitalized cases and 5.8 days (range, 1–9 days) for unhospitalized cases, and the mean time to discharge was 10.4 days (range, 1–15 days). The estimated mean serial interval was 13.7 days (range, 2–18 days). Estimates of the natural history are summarized in Table 2.

**Table 2:** Estimated key time periods (median, mean, SD, range) and number of observations (N)

	N	Median	Mean	SD	Range
Incubation	8	10.0	9.7	3.7	(6–15)
Symptom onset to hospital admission	15	4.0	4.5	2.6	(1–9)
Symptom onset to hospital discharge	5	15.0	10.4	6.5	(1–15)
Symptom onset to death in hospital	19	5.0	6.6	5.9	(0–22)
Symptom onset to death in community	4	6.5	5.8	3.9	(1–9)
Hospital admission to discharge	5	9.0	7.0	4.3	(3–12)
Hospital admission to death	18	2.0	3.1	3.1	(0–8)
Serial interval	12	15.0	13.7	4.5	(2–18)

### Transmission chain

Nine EVD cases were imported into Pujehun district from July to November 2014 (Fig. 1b). Only one imported case resulted in an outbreak, the remaining eight imported cases generated a total of three secondary cases (Fig. 1b). The main outbreak involved two close areas, namely Zimmi and Dumagbe, located in the Eastern part of the district, connected by family and commercial links; 39 out of 40 local transmission events were resolved. Six transmission events occurred in Zimmi and 27 in Dumagbe (Fig. 1b). The index case (patient ID: 1), a man escaped from the Ebola Treatment Center in Kenema and travelling to Zimmi, developed clinical symptoms on July 7, 2014, and died on July 11, 2014, without being isolated in Pujehun EHC and consequently infected two relatives (ID: 2 and 3) and one roommate (ID: 4), living in the same house as the index case in Zimmi. Patients 2 and 4 did not transmit the infection but patient 3 infected four persons, including the religious chief of Dumagbe (ID: 10) and one patient (ID: 11) who infected four persons, two of them living in Dumagbe (ID: 16 and 19). Thus, two different persons transmitted the infection in Dumagbe. Afterwards, the infection spread in Dumagbe in two separate chains of transmission exclusively through family contacts (Fig. 1b). The two transmission chains were sustained by few cases that infected three or four persons, while most of the cases did not transmit the infection (Fig. 1b). The number of secondary cases ranged from 0 to 4, and 65 % (32/49) of cases did not transmit the infection (Fig. 1b and c). The distribution of secondary cases is well fitted by a negative binomial distribution (*χ*^2^ = 8.83, *P* = 0.07) with dispersion parameter *k* = 0.45 (95 % CI, 0.19–1.32; Fig. 1c), while it does not comply with a Poisson distribution (*χ*^2^ = 31.11, *P* <0.001), the expected distribution in case of homogeneous transmission. The mean number of secondary cases in the first month of the outbreak (first 8 cases) was *R*_*0*_ = 1.63 (range, 0–4) (Fig. 1b). Overall, 74.3 % (29/39) of transmission events occurred between members of the same household or extended family (Fig. 1b,d), 17.9 % (7/39) of transmission events occurred in the community, mainly between friends (Fig. 1b and d), and 7.7 % (3/39) of transmission events were healthcare related (Fig. 1b,d). Specifically, one driver (ID: 36) was infected during the transport of two EVD cases from Zimmi to Pujehun EHC, one nursing aide (ID: 37) was infected in the Pujehun EHC, and one patient (ID: 49) was infected in Zimmi EHC (the first test was negative and the second, 11 days after discharge, resulted positive). It was possible to establish the type of contact with the primary infector for 31 case patients (Table 1).

### Reproduction numbers

**Fig. 2:**
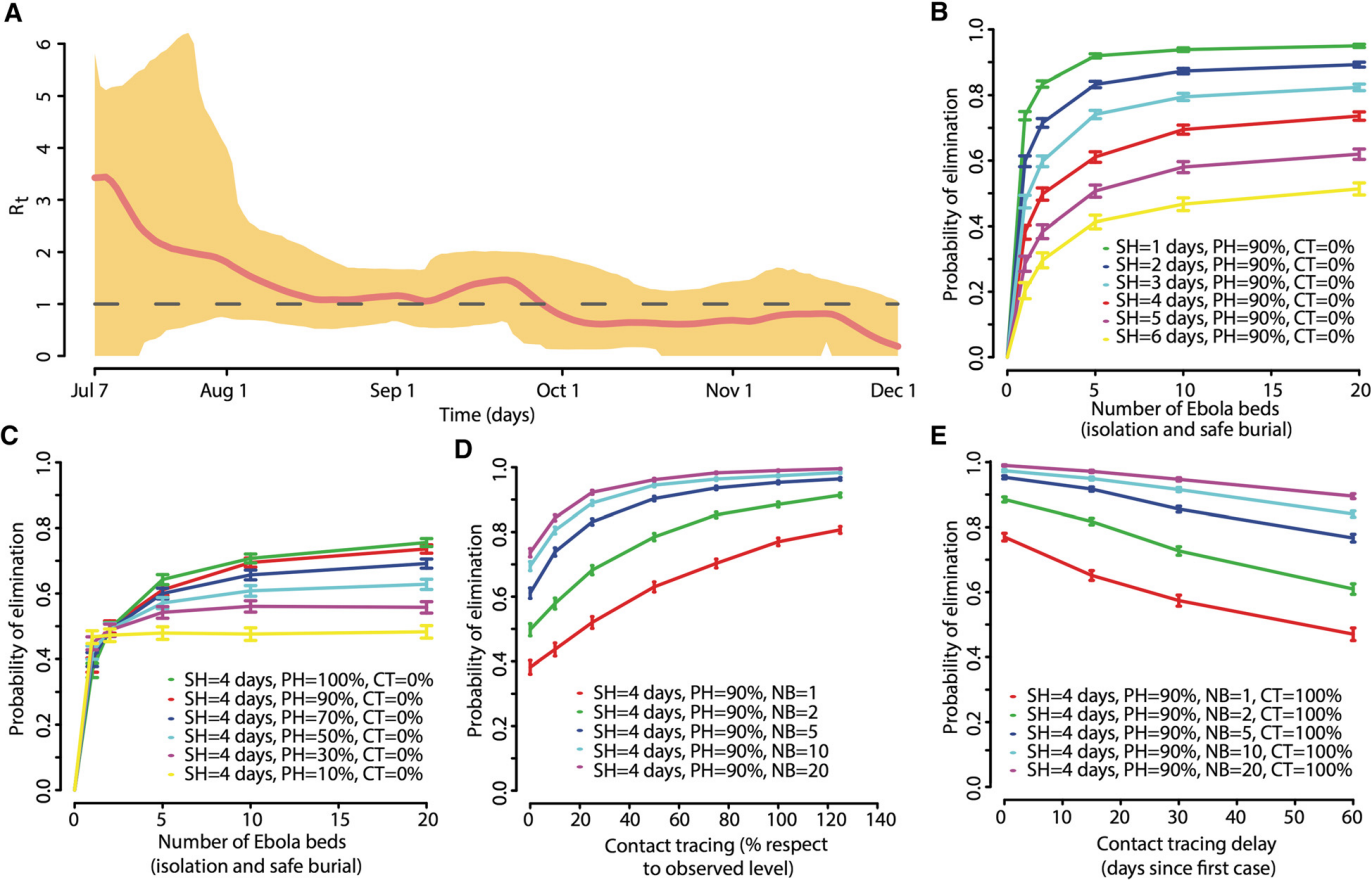
Impact of interventions in Pujehun district. The probability of eliminating an otherwise large outbreak is defined as 1–*p*_*i*_ */p*_*ni*_, where *p*_*i*_ and *p*_*ni*_ are the probabilities of outbreak with and without intervention [19]. (**a**) Net reproduction number over time. (**b**) Probability of disease elimination in Pujehun district by assuming isolation of cases and by varying the number of Ebola virus disease (EVD) beds and the average time from symptom onset to hospitalization in the absence of other interventions (e.g. safe burials for unhospitalized cases and contact tracing). Probability of hospitalization set to 90 %. (**c**) Probability of disease elimination in Pujehun district by assuming isolation of cases and by varying the number of EVD beds and the probability of hospitalization in the absence of other interventions (safe burials for unhospitalized cases and contact tracing). The average time from symptom onset to hospitalization is set to 4 days. (**d**) Probability of disease elimination in Pujehun district by assuming isolation of cases and contact investigation and by varying the contact tracing levels (with respect to observed values in Pujehun district) and the number of EVD beds. Contact tracing level is defined as the percentage of cases detected and isolated through contact investigation, namely 30 % in July 2014, 40 % in August, and 60 % in September. The average time from symptom onset to hospitalization is set to 4 days and the probability of hospitalization is set to 90 %. (**e**) Probability of disease elimination in Pujehun district by assuming isolation of cases and contact investigation and by varying the timing of implementation of contact investigation and the number of EVD beds. The contact tracing level is set to values observed in Pujehun district, namely 30 % in July, 40 % in August, and 60 % in September 2014. The average time from symptom onset to hospitalization is set to 4 days and the probability of hospitalization is set to 90 %

The model-based estimate of the basic reproduction number for Pujehun district in the absence of interventions was *R*_*0*_ = 2.24 (95 % CI, 1.52–4.51; Additional file 3). The mean net reproduction number showed a decreasing trend over time (Fig. 2a). The mean estimated for the first month of the epidemic was *R*_*t*_ = 2.24. *R*_*t*_ remained close to the elimination threshold from mid-August to mid-September 2014. After a raise of transmission in late September 2014, corresponding to the peak of deaths and discharges observed in the first week of October 2014 (Figs. 1a and 2a), *R*_*t*_ permanently remained below the elimination threshold.

### Interventions

The percentage of unhospitalized cases was 11.2 % (5/49). A total of 71.4 % (30/42) of fatal cases were buried the same day they died (maximum delay between death and burial: 2 days) and 11.9 % (5/42) of fatal cases were buried in the community. Community burials were recorded only at the very beginning of the outbreak (Fig. 1b). Suspected EVD cases were hospitalized in two distinct EHCs with a capacity of 20 EVD beds overall; the number of patients hospitalized at the same time was never above 10. About 250 contact tracers performed contact investigation; the mean number of contacts investigated per EVD case raised from 11.5 in July to 16 in August, reaching 25 in September 2014. The mean (±SD) number of confirmed cases among traced contacts of cases was 1.9 ± 2.2, corresponding to a percentage of 16.6 ± 22.5 %. Overall, at least 42.5 % (17/40) of case patients infected through local transmission events were detected by contact tracing, an underestimate of the true value as it was based on the analysis of 21 contact tracing forms only. By assuming that detection (42.5 %) is proportional to the mean number of contacts investigated per EVD case, we estimated that the percentage of cases detected through contact tracing might have increased from about 30 % in July to 40 % in August and reached 60 % in September 2014.

### Impact of interventions in Pujehun

By assuming that case isolation is the only implemented intervention measure (90 % probability of hospital isolation and 4 days on average from symptom onset to hospitalization), we found that the probability of eliminating an otherwise large outbreak increased from 38.2 % (95 % CI, 36.0–40.3) to 73.6 % (95 % CI, 72.3–74.9) by increasing the number of EVD beds from 1 to 20 (Fig. 2b). The time from symptom onset to hospitalization had a dramatic impact on the probability of disease elimination (Fig. 2b). No large differences were observed by varying the probability of hospitalization between 50 % and 100 % (Fig. 2c). The above probabilities increased to 77.0 % (95 % CI, 75.8–78.2) and 99.0 % (95 % CI, 98.8–99.2), respectively, by additionally assuming contact tracing at the levels observed in Pujehun district since the beginning of the outbreak (Fig. 2d). The above probabilities decreased to 47.0 % (95 % CI, 45.1–48.9) and 89.5 % (95 % CI, 88.8–90.2), respectively, by assuming that contact investigation starts 60 days later (Fig. 2e). The results did not change substantially by assuming the availability of only five EVD beds instead of 20. We did not find a significant impact of safe burial procedures among unhospitalized cases on the probability of disease elimination.

## Discussion

The current EVD epidemic in West Africa has been generated by many local asynchronous outbreaks at district level [8]. In this respect, the Pujehun outbreak represents a precious opportunity for understanding detailed dynamical insights into the transmission of EVD, both concerning its epidemiological features and the assessment of control measure effectiveness.

Our estimates of the key time periods are coherent with the literature [3, 9]. The incubation period (9.7 days on average) might be underestimated as it is defined as the time from the last contact with the infector to symptom onset, the only available information (Additional file 2). Unfortunately, we did not have information about potential previous contacts – this information was not collected, as the aim was to define the follow-up period for contacts of cases. More in general, it should be kept in mind that data were collected under extreme conditions and estimates might be affected by the missing data. As for the time from symptom onset to hospitalization, our estimate (4.5 days on average) is similar to that observed in Sierra Leone (4.6 days on average) and West Africa (5.0 days on average) in the same period [3]. However, the observed high hospitalization rate, close to 90 %, and the early implementation of containment measures suggest a higher detection and isolation of cases, possibly resulting in an overall decrease of transmission in the community. This is ascribable to the availability of an adequate number of isolation units – 20 EVD beds in the two EHCs – and to an aggressive local policy of contact tracing – 25 contacts investigated per EVD case on average in September 2014. As for comparison, the percentage of isolated EVD cases in West Africa during the same period was 52 % (as reported by the WHO on November 5, 2014), while the target set by the WHO by 1 December 2014 was 70 %.

Model simulations support the relevant role of isolation of cases and contact tracing. By assuming that containment measures are implemented from the very beginning of the outbreak, we estimate that the probability of disease elimination could have been as high as 99.0 % (95 % CI, 98.8–99.2) as a combined result of isolation of cases and contact tracing as performed in Pujehun district. We found that 74.3 % of transmission events occurred between members of the family (a percentage about three times higher than that of influenza [10, 11]) or extended family and most of the remaining transmission occurred between friends – a pattern similar to that found in Guinea [12] and ascribable to the fact that EVD is mainly transmitted through unprotected physical contacts. Therefore, contact investigation could have been facilitated by close social and demographic relationships between cases.

Additional support comes from our estimates of the reproduction number. Our estimates (1.63 from the analysis of the transmission chain; 2.24 from the analysis of incidence) comply with those provided by other groups [3, 13–16], although it is very critical to compare estimates of *R*_*0*_ for different geographical regions as they strongly depend on several factors, including individual behavior and control measures in place, among others. Estimates of the serial interval are also similar to those provided by other groups [3] and of other hemorrhagic fevers, e.g. Marburg [17]. However, our estimates of the net reproduction number show a rapid decrease to values close to the elimination threshold less than 2 months after the first case. Elimination could have been facilitated by the overdispersed distribution of the number of secondary cases (*k* = 0.45, 65 % of cases did not transmit the infection) – it is well known that the probability of outbreak and elimination depends on the distribution of secondary cases per single primary case [18, 19] – and by the formation of clusters in the beginning of the outbreak (with high *R*_*t*_ estimate).

Only one patient and two HCWs were infected in a hospital setting: this could have perhaps reflected adequate healthcare system preparedness, also witnessed by the early availability of 20 EVD beds and 250 contact tracers. The first local personnel training program started on April 2014, about 3 months before the first case registered. Indeed, a third HCW (one nurse of the Pujehun burial team) was infected during private treatment at the home of a friend, but we classified this event as transmission in the general community. None of the 74 Peripheral Health Units was closed during the outbreak and 10,285 childbirths were recorded in 2014 (without significant variations with respect to 2013, namely 9,657 childbirths). The relatively minor role of transmission during burial rituals appears to have been due to the aggressive local policy regarding funerals and the population’s reportedly very good compliance of such.

In Sierra Leone, the proportion of EVD cases reporting having attended a funeral within 1 month of symptom onset has decreased from about 30 % in May–September 2014 to less than 20 % in October 2014–January 2015 [20]. In Pujehun district, community burials could have contributed to infection transmission only in the very initial phase of the outbreak. Other factors could make containment even more challenging in different areas; herein, the outbreak spread in a very isolated geographical area, thus making it easier to prevent the spreading to other villages (e.g. EVD checks at roadblocks around Zimmi and Dumagbe). The small population size of the two most affected villages contributed to successful contact investigation, which is likely more challenging in an urban context.

## Conclusions

The most important factors affecting the probability of disease elimination are the number of EVD beds and the percentage of cases detected and isolated through contact investigation. These factors strongly depend on preparedness (such as rapid medical supplies and organization of contact investigation procedures), population awareness, and compliance with intervention policies. Our simulations suggest that rapid implementation of these measures would also play a critical role, especially in cases of a suboptimal number of EVD beds and low contact tracing levels. Overall, a timely and aggressive activation of countermeasures appears to be decisive in the prevention of EVD outbreak scale-up to national or international level.

In their recent article, Henao-Restrepo et al. [21] demonstrated an efficacy of 100 % (95 % CI, 74.7–100.0) of the EVD vaccine candidate rVSV-ZEBOV and suggest that it might be effective at the population level when delivered through a ring vaccination strategy (vaccine effectiveness at ring level around 75 %). Our findings are relevant to evaluate the effects of the vaccine in containing an emerging EVD outbreak, as the outcome of ring vaccination policies strongly depends not only on the number of available doses, population compliance and timing of vaccine administration, but also on the ability of detecting cases and tracing their contacts.

## Additional files

Additional file 1: contact tracing form. (PDF 65 kb) Additional file 2: patient records. (XLSX 44 kb) Additional file 3: additional methods. (PDF 2635 kb)

## Abbreviations

EHC: Ebola holding center
EVD: Ebola virus disease
HCW: Health care worker
