# Supplementary material for: The 2014 Ebola virus disease outbreak in Pujehun, Sierra Leone: epidemiology and impact of interventions
Source: BMC Med. 2015 Nov 26;13:281. doi: 10.1186/s12916-015-0524-z (PMC4660799; doi:10.1186/s12916-015-0524-z)
Supplement: Additional file 1: contact tracing form. — (PDF 65 kb) [file 12916_2015_524_MOESM1_ESM.pdf]

MINISTRY OF HEALTH AND SANITATION:. EBOLA VIRUS DISEASE CONTACT LINE LISTING FORM

CASE MANAGEMENT

Case ID	Surname	Other name	Head of Household	Village	Sub-Country	District	Date of Symptom onset	Date of admission to Isolation	Date of death			
001	X	Y	Z	Zimmi	Sierra Leone	Pujehun	07/07/14	-	11/07/14			

***For all information on location, please list information on where the contact will be residing for the next month

Name of contact	Sex (M/F)	Age (Yrs.)	Relation to case	Date of last contact with case	Type of contact (1,2,3,4)*List all	Head of Household	Village/Street	District	Village leader	Phone Number	Healthcare Worker (Y/N) if Yes, what facility?
A	F	48	Nice	10/07/14	3,4	Z	Zimmi	Pujehun	W		No
B	M	54	Brother	11/07/14	1,2	Z	Zimmi	Pujehun	W		No
C	M	49	Uncle	09/07/14	3,4	Z	Zimmi	Pujehun	W		No
D	F	85	Brother	10/07/14	3,4	Z	Zimmi	Pujehun	W		No
E	M	39	Uncle	11/07/14	3,4	Z	Zimmi	Pujehun	W		No
F	F	28	Father in Law	09/07/14	2,3	Z	Zimmi	Pujehun	W		No
G	F	8	G/Father	09/07/14	3,4	Z	Zimmi	Pujehun	W		No

Types of contact: 1= Touched the body fluids of the case(blood, vomit, saliva, urine, faeces); 2= Had direct Physical contact with the body of the case (alive or dead); 3= Touched or shared the linens, clothes, or dishes/eating utensils of the case; 4= Slept, ate, or spent time in the same household or room as the case
