# Supplementary material for: The 2014 Ebola virus disease outbreak in Pujehun, Sierra Leone: epidemiology and impact of interventions
Source: BMC Med. 2015 Nov 26;13:281. doi: 10.1186/s12916-015-0524-z (PMC4660799; doi:10.1186/s12916-015-0524-z)
Supplement: Additional file 3: additional methods. — (PDF 2635 kb) [file 12916_2015_524_MOESM3_ESM.pdf]

The 2014 EVD outbreak in Pujehun, Sierra Leone: epidemiology and impact of interventions.

Marco Ajelli[1], Stefano Parlamento[1], David Bome[2], Atiba Kebbi[3], Andrea Atzori[4], Clara Frasson[4], Giovanni Putoto[4], Dante Carraro[4], Stefano Merler[1*]

[1]Bruno Kessler Foundation, Trento, Italy
[2]District Medical Officer, Pujehun District, Ministry of Health and Sanitation, Sierra Leone
[3]Medical Superintendent Pujehun Hospital, Ministry of Health and Sanitation, Sierra Leone
[4]Doctors with Africa - CUAMM, Italy

[*]corresponding author; E-mail: merler@fbk.eu.

Supplementary Information

Contents

1 Transmission model and Ebola natural history **2**

2 Interventions **3**

3 Markov chain Monte Carlo calibration **4**

4 Reproduction numbers **5**

5 Superspreaders **7**

Bibliography **8**

1 Transmission model and Ebola natural history

Each individual of the population of the Pujehun district of Sierra Leone (about 350,000 individuals) is explicitly simulated as an agent of the individual-based model. Individuals are grouped in households according to the DHS data[1], and households are grouped in villages geographically located in the Pujehun district.

The progression of EVD is modeled by adopting the epidemic model originally introduced by Legrand et al.[2]: susceptible individuals (S) can acquire infection after contact with an infectious individual and become latent (asymptomatic and not able to transmit the infection), E; at the end of the latent period (which is assumed equal to the incubation period as pre-symptomatic transmission is very unlikely to occur and it has not been proved for EVD yet), infectious (symptomatic) individuals, I, can transmit infection to both household members and members of the additional households (e.g. extended family). Infectious individuals at home then may either be hospitalized (H), die (D) or recover (R). Hospitalized individuals may either die or recover. After recovery, hospitalized individuals remain in the hospital (though no longer infectious) for an additional period. Deceased individuals may transmit infection during their funeral (to household members and to the general community) and are then removed (R). The duration of each key time period used in the model, case fatality rate, and hospitalization rate are reported in the main text and have been derived from the analysis of the data collected in this study (see Fig. S1).

As described above, the model accounts for two main routes of transmission: transmission in households and in the general community (to additional households) when the infected individuals are not hospitalized, and transmission during funerals (to household and additional household members). In fact, as suggested by the analyzed data (see main text) we assume that hospitalized individuals are efficiently isolated and thus not able to transmit the infection (only 7.7% of transmission events occurred in hospital).

Simulations were initialized with 1 (randomly selected) infected individuals, at the time corresponding to the first case imported in the Pujehun district (see main text).

Transmission within households As in Merler et al[3], the transmission is assumed to be homogeneous between members of the same household. At time t, a non-hospitalized infectious individual j transmits EVD to all other members of her or his household with the following force of infection:

$$\lambda_j(t) = \frac{\beta \rho_j}{N_{f_j}(t)} \tag{1}$$

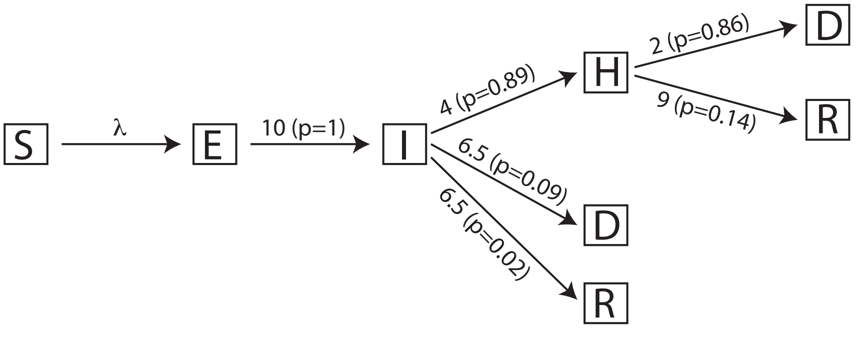

Figure S1: EVD transmission model. For each transition, the median duration (in days) and the probability of occurrence are shown.

where β is the transmission rate in households (assumed to be the same for all households), $N_{f_j}(t)$ is the household size at time t (thus excluding deceased and hospitalized members), and ρ_j is the infectiousness of individuals j, which is sampled from a gamma distribution (see Sec. 5).

Transmission in the general community Each household is linked to five additional households, which are sampled from households within 10 km proportionally to the population density to account for contacts occurring in the general community that, in the case of EVD, mainly correspond to contacts between relatives (for instance for taking care of sick individuals). (A sensitivity analysis of the results obtained by varying these assumptions have already been presented in Merler et al.[3]). Let a_j be the set of additional households for an infectious (non–hospitalized) individual j. Note that a_j does not include the household of individual j and thus does not include j. Individual j transmits the infection to the members of a_j with force of infection:

$$\lambda_j(t) = \frac{\sigma\beta\rho_j}{N_{a_j}(t)} \tag{2}$$

where $N_{a_j}(t)$ is number of individuals in a_j at time t and σ is the ratio of the transmission rate in the additional households compared to that in the household of individual j $(0 \leq \sigma \leq 1)$.

Transmission during burial ceremonies During unsafe burials, a deceased individual j transmits EVD to her/his household members and members of the additional households a_j similarly to transmission in households, namely:

$$\lambda_j(t) = \frac{\beta\rho_j}{N_{f_j}(t)} \tag{3}$$

to household members and

$$\lambda_j(t) = \frac{\sigma\beta\rho_j}{N_{a_j}(t)} \tag{4}$$

to members of the additional households. Note that the same set of households involved in the general community transmission is assumed also for burial ceremonies[3].

Overall force of infection and probability of getting infected At any time t of the simulation (we consider a time step $\Delta t = 1$ day), any susceptible individual i has a probability $p_i(t) = 1 - \exp\left(-\Delta t \sum_j \lambda_j(t)\right)$ of being infected from each infectious individual j in the population.

2 Interventions

According to our investigation, three intervention measures have been performed in the Pujehun district during the 2014 Ebola outbreak: isolation of cases admitted to the hospital, community safe burials, and contact tracing.

Suspected Ebola cases admitted to hospital have been isolated from non-Ebola patients (see main text). This is simulated in the model by assuming no Ebola transmission in hospitals.

Accoring to the data reported in the main text, 50% of Ebola cases who died before August 17 were safely buried; after that all cases were safely buried. This is simulated in the model by assuming that individuals dieing before August 17, have 50% probability of being safely buried, while this probability becomes 100% for the rest of the outbreak; no Ebola transmission is assumed to occur during safe burial procedure.

Once an Ebola case has been admitted to the hospital, contact investigation starts. The average number of traced contacts for admitted case has increased over the course of the outbreak and the total number of cases identified through contact investigation was about 43% (see main text). In the model we

assume than once a case is admitted to the hospital an increasing fraction of her/his contact is followed (assuming the same increment as observed in the data) in such a way that the cumulative number of identified cases through contact investigation is 43%. Moreover, we assume that once a case is identified through contact investigation it is effectively isolated and she/he is thus not able to transmit the infection.

A detailed sensitivity analysis of the effect of timing and intensity of the different interventions (such as time between symptom onset and admission to hospital, hospitalization probability, number of hospital beds reserved for Ebola patients, safe burial probability, number of traced contacts over time) has been performed.

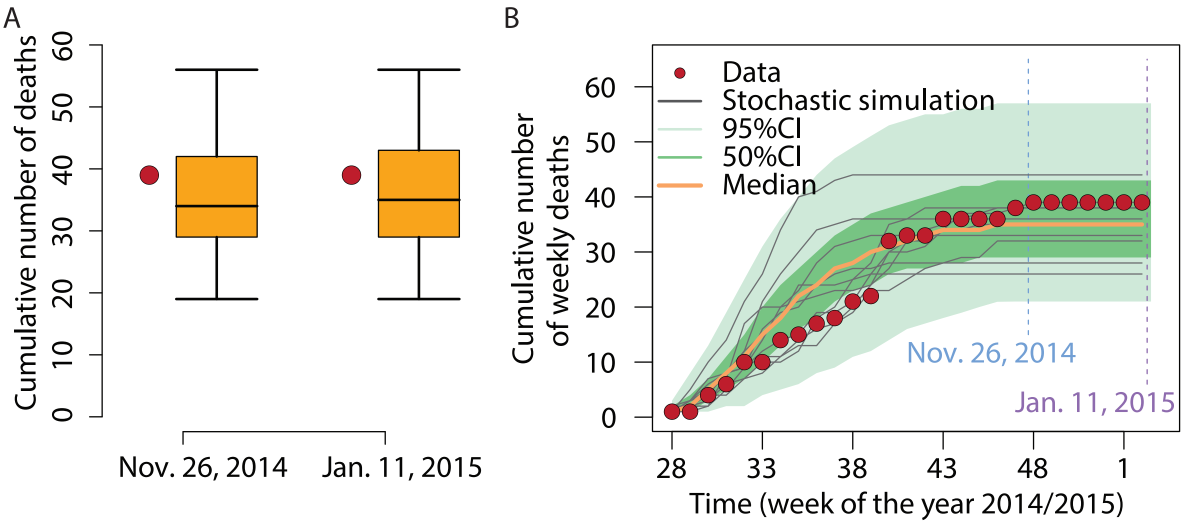

Figure S2: **A** Cumulative number of deaths in the Pujehun district as of November 26, 2014 and as of January 11, 2015 (corresponding to the day when the WHO declared the Pujehun district to be Ebola free). Red points correspond to the observed data, the orange boxes show 2.5%, 25%, 50%, 75%, and 97.5% quantiles of model simulations. **B** Observed cumulative number of deaths in Pujehun district (red points), 95%CI of model simulations (light green areas), 50%CI of model simulations (dark green areas) and median (orange). Grey lines represent 10 randomly selected model simulations.

3 Markov chain Monte Carlo calibration

The model has two unknown parameters, both related to virus transmissibility: β and σ (see Sec. 1). We do not have specific knowledge on the prior distribution of the two parameters; we therefore assume the prior distributions of β to be uniform in the interval $[0, 100]$, and for σ to be uniform in the interval $[0.1, 1]$. The lower bound of the prior distribution of σ was chosen in such a way to guarantee that a fraction of cases must occur outside the household members in order to be compliant with the findings presented in the main text. The posterior distribution of the parameter vector $\Theta = (\beta, \sigma)$ was explored by Markov chain Monte Carlo (MCMC) sampling applied to the likelihood of the recorded number of deaths in the population.

Specifically, by assuming the number of deaths to be Poisson distributed around the mean and independent for the two time intervals considered, we can write the total likelihood as $L = \prod_{i=1}^{2} P(w_i(\Theta); k_i)$, P is the probability of observing k_i events from a Poisson distribution with mean $w_i(\Theta)$, where k_i is the

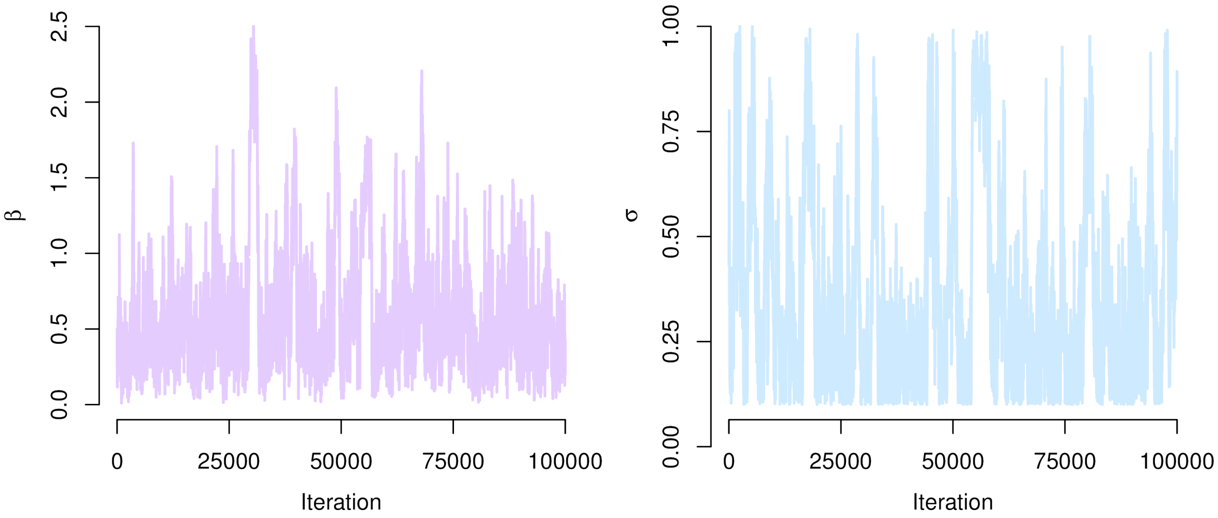

Figure S3: Estiamted value of β (left panel) and σ (right panel) at each time step of the MCMC procedure.

observed number of deaths in time interval i, and $w_i(\Theta)$ is the predicted number of deaths in time interval i (assuming the candidate parameter vector Θ).

Random-walk Metropolis-Hastings sampling is used to estimate Θ. At each iteration, the likelihood of a new candidate vector of parameters is evaluated and the candidate is either accepted or rejected following the usual Metropolis-Hastings algorithm.[4] As simulations are stochastic, whenever new candidate vectors are not accepted 10 times in row, the likelihood of the current parameter set is re-evaluated and the new likelihood accepted with probability 1[5]. This ensures that the chain does not remain trapped in a local maximum. The values of β and σ of a new candidate parameter vector are randomly sampled from normal distributions having mean equal to the current transmission rate and variance δ^2[6].

The two values of δ are chosen in such a way to guarantee a good acceptance rate. We perform 100,000 iterations. We check convergence by considering several different starting points and by visual inspection. We discard the first 10,000 iterations as burn-in period and, in order to get a subset of independent samples, we consider one sample every 10 of the remaining iterations. The reported mean values and confidence intervals are then computed from these stochastic realizations of the model, and thus account for both the stochasticity of model realizations and the uncertainty in model parameters estimates.

Fig. S2 shows a comparison between observed and simulated number of deaths. Fig. S3 shows the value of β and σ resulting at each iteration of the MCMC procedure; the estimated mean values of the two parameters are $\beta = 0.619$ (95%CI: 0.134 – 1.7195) and $\sigma = 0.358$ (95%CI: 0.107–0.901).

4 Reproduction numbers

Model based estimate of the basic reproduction number The basic reproduction number R_0 is defined as the average number of secondary infections caused by a typical infective in a fully susceptible population.[7] A relation exists between R_0 and the generation time (i.e., the time elapsing between infection of a primary case and infection of a secondary case caused by the primary case) and the exponential growth rate of the epidemic.[8]

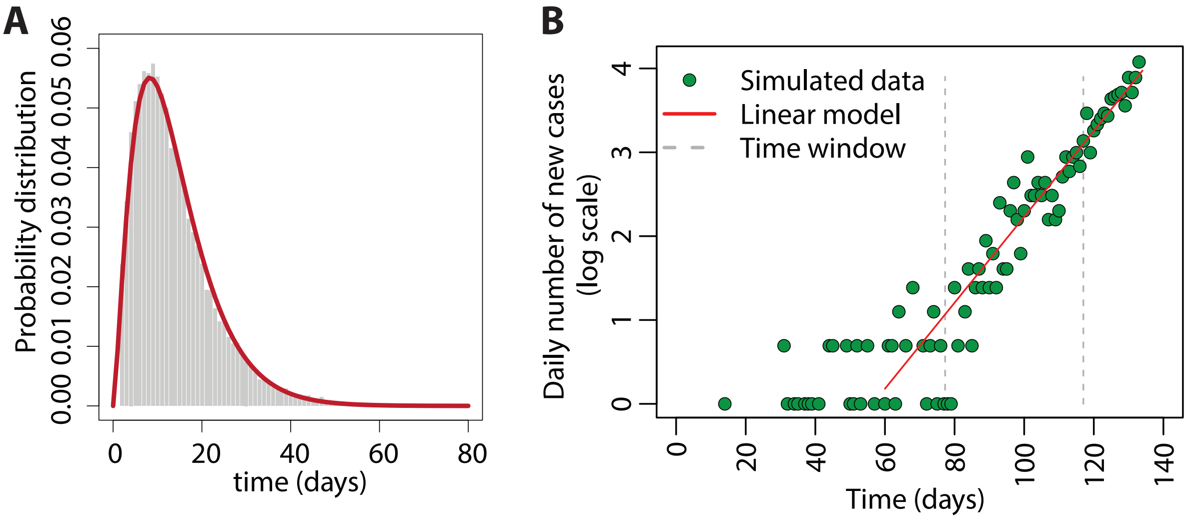

Figure S4: **A** Probability distribution of the generation time. Grey ears represent the simulated generation time in the model (directly computed by the differences between the times of infection of infectors and infectees); red line represents the gamma distribution maximizing the likelihood. **B** Model estimate of R_0 for one stochastic simulated EVD epidemic in the absence of interventions in Pujehun district. The time window for the computation of the exponential growth rate r is set to 40 days, i.e. three generation times.

In the case of an exponentially distributed generation time, this relation is simply given by $R_0 = 1 + rT_g$, where r is the exponential growth rate of the simulated epidemic during the initial phase (when the depletion of susceptible individuals is negligible) and T_g is the estimated generation time.

For generic distributions of T_g, we use the following procedure[9–14]. First of all, we calculate the generation time empirically by keeping track of it in model simulations, and we fit (maximum likelihood) an appropriate distribution to the resulting generation time frequencies (a Gamma distribution in our case). Then, the basic reproduction number can be computed as:

$$R_0 = \frac{1}{\int_0^{\infty} \hat{\gamma}(\tau) e^{-r\tau} d\tau}$$

where $\hat{\gamma}(\tau)$ is the fitted Gamma distribution for the generation time.

Model based estimates of R_0 are obtained by simulating EVD epidemics with transmission parameters estimated by the MCMC procedure but assuming no interventions (we recall that transmission parameters are estimated by considering all interventions performed in Pujehun district since the beginning of the outbreak). This gives raise to simulated epidemic characterised by clearly visible exponential growth in the first phase, and thus it is possible to estimate the exponential growth rate r of simulated epidemics (see Fig. S4B). By keeping track of the time from infection in primary infectors and infection in secondary cases, we estimate a generation time of 13·9 days (SD: 9·3 days), which is fitted by a Gamma distribution of parameters shape 2·46 (SE: 0·013) and rate 0·176 (SE: 0·0011), see Fig. S4A. As r is computed for each stochastic realization of the model, the resulting R_0 accounts for both the stochasticity of the simulations and the transmission rates obtained from the Markov chain Monte Carlo procedure.

Net reproduction number By assuming that the time of infection is known for of all cases, i.e. the number of daily cases $C(t)$ at time t is known, the net reproduction number over time can be estimated by approximating $C(t)$ by a Poisson process:

$$C(t) \approx Pois(R_t \sum_{s=1}^{t} T_g(s)C(t-s))$$

where T_g is the generation time distribution, and R_t is the instantaneous reproduction number at time t. The likelihood is therefore

$$L = \prod_{t=1}^{T} P(C(t), R_t \sum_{s=1}^{t} T_g(s)C(t-s))$$

where $P(k, \lambda)$ is the probability mass function of a Poisson distributions (i.e., the probability of observing k events if these events occur with a known rate λ). R_t can be estimated by maximum likelihood.

The time of infection is known only for a subset of cases. We assigned the time of infection to other cases by using the following approximation. Let us consider a case and assume that the time of infection is not known but time of symptom onset t_s is. The time of infection is approximated as $t_i = t_s - \Gamma(l)$, where $\Gamma(l)$ is a random number form a Gamma distribution of mean $l = 9.7$ days (i.e. our estimate of the incubation period). A similar procedure can be used to assign the time of infection to all cases. This procedure allows estimating an instance of R_t. Means and confidence intervals of R_t were estimating by repeating 10,000 times the above procedure.

5 Superspreaders

A characteristic feature of the current Ebola epidemic is the heterogeneity in infectiousness of different individuals[15,16], i.e., a small fraction of infected individuals is responsible for the large majority of secondary cases. By analyzing the transmission chain reported in Fig. 1b of the main text we inferred the distribution of secondary cases generated by each Ebola case. By fitting this data with a negative binomial distribution (see Fig. 1 of the main text), we found that the distribution yielding maximum likelihood has mean $\mu = 0.79$ and dispersion $k = 0.45$, which is in less dispersed than that estimated for Guinea - $k = 0.18$[15].

Following the arguments presented in[17], we modeled the heterogeneity in infectiousness by multiplying the transmission rates by a factor ρ_i (see equations 1–4), which is sampled from a gamma distribution with shape 0.45 (i.e., the dispersion parameter k of the estimated negative binomial distribution) and scale 1.77 (i.e., $(1-p)/p$, where p is the probability of the estimated negative distribution, which can be computed as $k/(k+\mu)$).

References

[1] The DHS Program. Demographic and Health Survey 2007, 2007.

[2] J. Legrand, R. F. Grais, P.-Y. Boelle, A.-J. Valleron, and A. Flahault. Understanding the dynamics of Ebola epidemics. *Epidemiology and infection*, 135(04):610–621, 2007.

[3] Stefano Merler, Marco Ajelli, Laura Fumanelli, Marcelo FC Gomes, Ana Pastore y Piontti, Luca Rossi, Dennis L Chao, Ira M Longini, M Elizabeth Halloran, and Alessandro Vespignani. Spatiotemporal spread of the 2014 outbreak of Ebola virus disease in Liberia and the effectiveness of non-pharmaceutical interventions: a computational modelling analysis. *The Lancet Infectious Diseases*, 15(2):204–211, 2015.

[4] W. R. Gilks. *Markov Chain Monte Carlo*. Wiley Online Library, 2005.

[5] S. Cauchemez, A.-J. Valleron, P.-Y. Boelle, A. Flahault, and N. M Ferguson. Estimating the impact of school closure on influenza transmission from Sentinel data. *Nature*, 452(7188):750–754, 2008.

[6] S. Merler and M. Ajelli. Deciphering the relative weights of demographic transition and vaccination in the decrease of measles incidence in Italy. *Proc R Soc B*, 281(1777):20132676, 2014.

[7] R. M. Anderson and R. May. *Infectious diseases of humans*. Oxford university press Oxford, 1991.

[8] J. Wallinga and M. Lipsitch. How generation intervals shape the relationship between growth rates and reproductive numbers. *Proc R Soc B*, 274(1609):599–604, 2007.

[9] WHO Ebola Response Team. Ebola Virus Disease in West Africa — The First 9 Months of the Epidemic and Forward Projections. *N Eng J Med*, 371:1481–1495, 2014.

[10] S. Merler and M. Ajelli. The role of population heterogeneity and human mobility in the spread of pandemic influenza. *Proc R Soc B*, 277(1681):557–565, 2010.

[11] N. M. Ferguson, D. A. T. Cummings, C. Fraser, J. C. Cajka, P. C. Cooley, and D. S. Burke. Strategies for mitigating an influenza pandemic. *Nature*, 442(7101):448–452, 2006.

[12] M. Ajelli and S. Merler. The impact of the unstructured contacts component in influenza pandemic modeling. *PLOS ONE*, 3(1):e1519, 2008.

[13] G. Chowell, N. W. Hengartner, C. Castillo-Chavez, P. W. Fenimore, and J. M. Hyman. The basic reproductive number of Ebola and the effects of public health measures: the cases of Congo and Uganda. *J Theor Biol*, 229(1):119–126, 2004.

[14] Marco Ajelli and Stefano Merler. Transmission potential and design of adequate control measures for Marburg hemorrhagic fever. *PloS one*, 7(12):e50948, 2012.

[15] C L Althaus. Ebola superspreading. *Lancet Infect Dis*, 15(5):507–508, 2015.

[16] O Faye, PY Boëlle, E Heleze, O Faye, C Loucoubar, N Magassouba, B Soropogui, S Keita, T Gakou, el HI Bah, L Koivogui, AA Sall, and S Cauchemez. Chains of transmission and control of Ebola virus disease in Conakry, Guinea, in 2014: an observational study. *Lancet Infect Dis*, 15(3):320–6, Mar 2015.

[17] J O Lloyd-Smith, S J Schreiber, P E Kopp, and W M Getz. Superspreading and the effect of individual variation on disease emergence. *Nature*, 438(7066):355–359, 2005.
